# Effect of resistance training on heart rate variability of anxious female college students

**DOI:** 10.3389/fpubh.2022.1050469

**Published:** 2022-12-01

**Authors:** Ran Li, Runsheng Yan, Weihao Cheng, Hong Ren

**Affiliations:** Sport Science School, Beijing Sport University, Beijing, China

**Keywords:** resistance training, anxious female college students, heart rate variability, autonomic nervous function, randomized controlled trial

## Abstract

**Introduction:**

Female college students are a group with high incidence of anxiety, and anxiety will lead to the disorder of autonomic nervous system (ANS), which will adversely affect their study and life. Resistance training plays a positive role in improving anxiety, but there is little evidence on whether resistance training can improve ANS of anxious female college students. Heart rate variability (HRV) has gained widespread acceptance in assessing ANS modulation. Therefore, the objective of this study aimed to investigate the effects of resistance training on heart rate variability (HRV) in anxious female college student.

**Methods:**

A randomized controlled study of resistance training intervention was conducted in 27 anxious female college students that assigned randomly into an intervention group (*n* = 14) and a control group (*n* = 13). The intervention group was intervened by cluster training for 8 weeks. Self-rating anxiety scale (SAS) was used. ANS is evaluated by short-term HRV. Muscle strength was assessed by 1 RM indirect method. Independent-sample *t*-test was used to test post-test–pre-test scores between the intervention and control groups.

**Results:**

After the intervention, SAS score of the intervention group was significantly decreased (*P* < 0.05), SDNN of the intervention group was significantly increased (*P* < 0.05) and LF/HF was significantly decreased (*P* < 0.05).

**Conclusion:**

The resistance training intervention adopted in this study significantly increased the HRV of anxious female college students and improved their autonomic nervous disorder.

## Introduction

With the continuous development of society and increasingly fierce social competition, college students are facing great pressure of study and life, and have become a high-risk group of anxiety. The incidence of anxiety among college students is as high as 54.4%, which is one of the common psychological problems faced by college students (1). Evidence-based research proves that female college students are more likely to be anxious (2, 3). However, anxiety is not a simple psychological problem, especially it will lead to the disorder of autonomic nervous system (ANS) that generally involves failure of sympathetic and parasympathetic nervous system. Heart rate variability (HRV) has gained widespread acceptance in assessing ANS modulation. Adolescent females with anxiety show a decrease HRV compared with healthy controls (4–6). Prospective studies have found that HRV reduction is an independent risk factor for cardiovascular disease risk and increased all-cause mortality (7). Moreover, the decrease of HRV is also an independent risk factor for metabolic diseases such as diabetes (8). Therefore, if the autonomic nervous dysfunction of anxious female college students is not corrected and intervened. Then, it will not only bring adverse effects to the current study and life, but also increase the risk of cardiovascular disease.

Medicine and cognitive behavioral therapy are effective methods to improve anxiety, but medicine usually has obvious side effects and is easy to cause excessive mental dependence (9). Cognitive intervention usually needs trained experts to complete (10). In contrast, exercise, as a safe and low-cost intervention method, can also improve anxiety (11), and at the same time, it can bring more health benefits, such as the improvement of cardiopulmonary endurance and muscle strength, etc. Evidence-based studies have shown that both aerobic exercise and resistance training can effectively improve HRV (12, 13). Compared with aerobic exercise, resistance training is more flexible and less dependent on training places. Resistance training intervention should be more acceptable to improve ANS. Interestingly, recent studies found that resistance training leads to improvement in cardiac autonomic control of patients with chronic metabolic diseases rather than healthy individuals (13). However, there is still a lack of evidence that resistance training intervention can improve ANS of anxious individuals. Therefore, the purpose of this study was to explore the influence of resistance training on the HRV of anxious female college students.

## Materials and methods

### Subjects

A total of 27 anxious female college students (18–25 years old) from non-training majors in Beijing Sport University were enrolled in the randomized controlled trial (Table 1). The included criteria were: (1) SAS score ≥ 50, (2) no cardiovascular diseases and other contraindications to exercise, (3) no exercise habits. The experiment was approved by the Ethics Committee of Beijing Sport University and followed the principles of the last revised Declaration of Helsinki (7^th^ revision of October 2013). All participants volunteered to participate in the experiment. Before the experiment started, they fully understood the content and process of the experiment and signed the informed consent form.

**Table 1:** Baseline characteristics of participants in intervention and control groups.

**Characteristic**	**Intervention**	**Control**
*n*	13	14
Age (years)	22.6 ± 2.5	22.5 ± 2.0
Height (cm)	163.5 ± 4.6	164.8 ± 3.6
Weight (kg)	58.3 ± 8.0	56.3 ± 5.9
BMI (kg/m^2^)	21.8 ± 2.7	20.7 ± 2.0
SAS	58.2 ± 6.2	54.7 ± 6.5

BMI, body mass index; SAS, Zung's self-rating anxiety scale.

### Study design

In this randomized controlled trial, the sample size was estimated based on the measurement data of HRV indices in previous study (14, 15). When Type I error was 5% (α = 0.05), Type II error was 80% (β = 0.20), respectively, to detect a 25% mean difference at end of intervention. The sample size required was calculated according to the formula *n*1 = *n*2 = 2(*Z*_α/2_ + *Z*_β_)^2^δ^2^/σ^2^. Therefore, the intervention group and the control group had about 13 subjects, respectively.

We conducted an 8-week randomized controlled trial. After completing the baseline measurements, the participants were randomly divided into intervention group (*N* = 13) and control group (*n* = 14) using a computer-generated simple randomization software. All participants were instructed to maintain their usual lifestyle habits and not to be engaged in other structured exercise interventions.

#### Training intervention

The intervention group was intervened by cluster training for 8 weeks. Cluster training is a special resistance training method, which is different from the traditional resistance training in the intermittent arrangement between work and rest. Under the same amount and intensity of training, the degree of autonomic nervous fatigue caused by cluster training is lower (16). The intermittent arrangement adopted in this experiment is to rest for 90 s between groups and rest for 30 s within groups. The training frequency is about twice a week, and the interval between two trainings is about 72 h. Each training session consists of three movements, including barbell bench press (pectoralis major), Lat pull-down machine; (latissimus dorsi) and leg lift machine (quadriceps femoris), with five groups of exercises for each movement. Training intensity is 70% 1 RM. Each training session starts with a 5-min warm-up exercise (low-intensity aerobic exercise), about 40 min of resistance training, and relaxation training for 5 min.

### Measurements

In the intervention, HRV indicator was the primary outcome. Muscle strength and anxiety level were the secondary outcomes.

#### HRV indicator evaluation

Subjects came to our laboratory, avoiding any physical activity since they woke up, between 7.00 and 9.00 a.m. following study pre-conditions: (1) fasting conditions; (2) not altered sleep pattern the night before; (3) to be abstained from alcohol intake and drugs or stimulant consumption, including coffee and other stimulants 24 h before; and (4) to avoid moderate-intensity physical activity within 24 h and vigorous-intensity physical activity within 48 h before the test. After the subjects arrived at the test site, they sat comfortable chair and rested for 5 min, then wore the heart rate meter in their left hand and the heart rate belt to the xiphoid process of sternum, and collected the R-R interval signals for 10 min in a quiet environment at thermo-neutral conditions (22–24°C and 40–60% relative humidity). The Polar heart rate (V800, Finland) was used to collect the R-R interval signals. Kubios HRV Standard 3.4 software (University of Eastern Finland, Kuopio, Finland) was used to calculate the HRV time domain and frequency domain indicators. Time domain indexes include SDNN (standard deviation of all RR intervals, ms) and RMSSD (square root of the sum of the mean of the difference between adjacent RR intervals, ms). Frequency domain indicators include low frequency power (LF, 0.04–0.15 Hz; sympathetic activity index) and high frequency power (HF, 0.15–0.40 Hz; vagus nerve activity level index) and LF/HF ratio (sympatheticvagus nerve balance index). The corrections to be made on the RR series are displayed on the RR interval axis. When the corrections are applied, detected artifact beats are replaced using cubic spline interpolation (17). HRV analyses were conducted by the same trained researcher to obtain reproducible and valid data.

#### One repetition maximum prediction

The 1 RM indirect test was used to evaluate the muscle strength of the subjects. Before the test starts, the subjects completed 8–12 lifts with light weight to get familiar with the movements and warm up fully, then chose a weight that can enable the subjects to complete 3 repetitions. If the repetitions exceeded 3 times, the subjects should have a rest for 2 min, and then increased the weight by 5% for the next set. This was repeated until the subject could finish exactly 3 repetitions, and the lifted weight and the number of repetitions were recorded and substituted into the formula: 1 RM = [lifted weight× (1 + 0.025× repetitions)] to estimate 1RM (18).

#### Measurement of anxiety level

Zung's Self-rating Anxiety Scale (SAS) was used to measure the anxiety degree of the subjects. The Chinese version of SAS has good reliability and validity (19). There are 20 questions in SAS. Each question is divided into 4 grades according to its severity, with scores of 1, 2, 3, and 4. The higher the score, the higher the degree of anxiety. The total score was multiplied by 1.25 and converted into a standard score. SAS standard score ≥50 was rated as anxiety.

### Statistical analysis

All experimental data were expressed as mean ± standard deviation and statistically analyzed by SPSS 17.0 (SPSS Inc., Chicago, IL, USA). The differences in HRV parameters, 1 RM and SAS score before and after the experiment were calculated, respectively, and independent samples *t*-test was used to test post-test–pre-test scores between intervention group and control group. In all cases, values of *P* < 0.05 were considered statistically significant.

## Results

**Figure 1:**
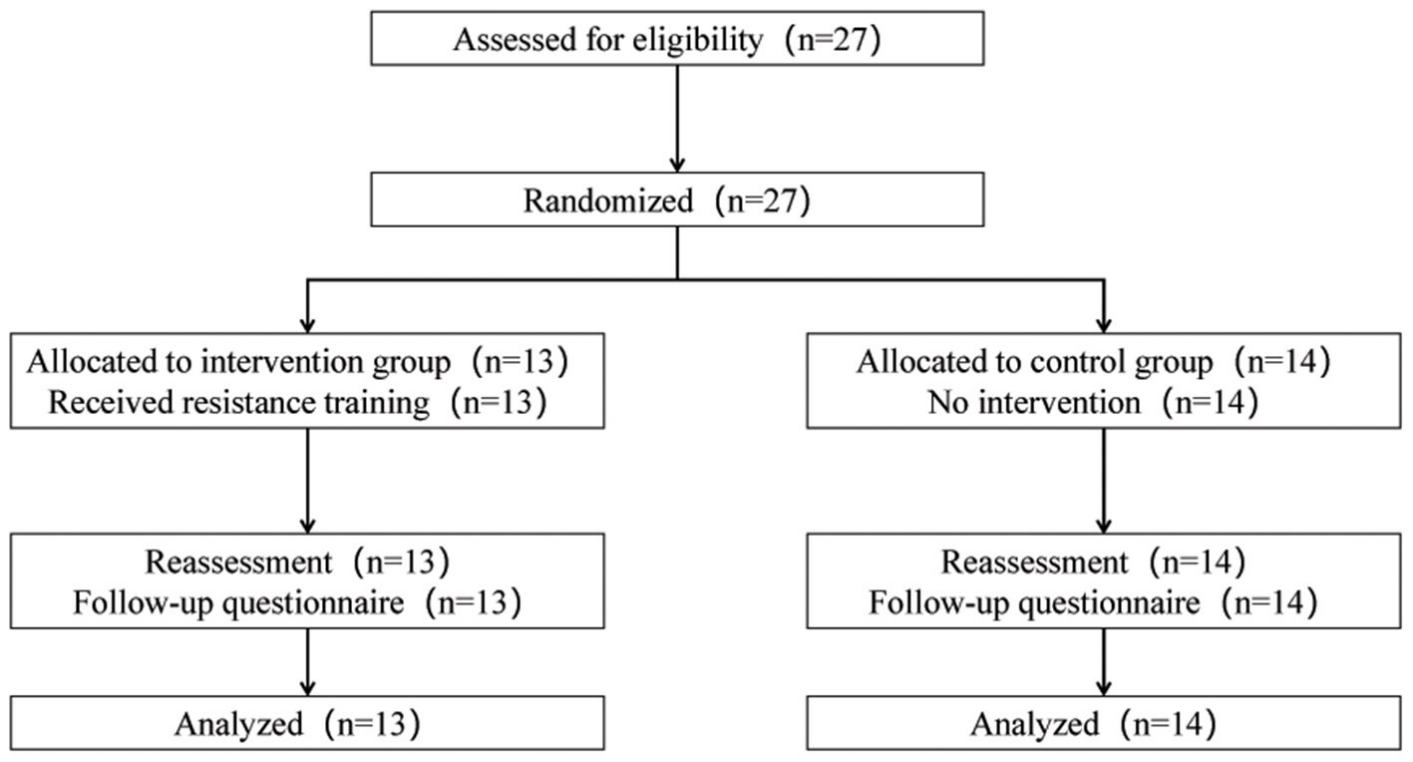
The flow of participants through the trial.

Figure 1 shows the flow of participants through the study. The baseline primary and secondary outcomes of all participants are described in Table 2. No differences were observed in the baseline values between intervention group and control group.

**Table 2:** Baseline primary and secondary outcomes of participants in intervention and control groups.

**Outcomes**	**Intervention**	**Control**	***T*-value**	***P*-value**
**Primary outcomes**				
SDNN (ms)	33.9 ± 9.2	36.5 ± 9.2	−0.725	0.475
RMSSD (ms)	32.1 ± 10.9	31.3 ± 12.0	0.167	0.868
PNN50 (%)	12.1 ± 10.6	13.8 ± 12.5	−0.385	0.704
LF (log)	6.2 ± 0.6	6.3 ± 0.6	0.062	0.951
HF (log)	5.7 ± 0.6	5.9 ± 0.8	0.392	0.698
LF/HF	2.2 ± 1.8	2.0 ± 1.5	0.369	0.715
**Secondary outcomes**				
BMI (kg/m^2^)	21.8 ± 2.7	20.7 ± 2.0	1.15	0.262
SAS	58.2 ± 6.2	54.7 ± 6.5	1.406	0.172
Barbell bench press (kg)	23.8 ± 5.8	22.5 ± 5.7	0.603	0.552
Lat pull-down (kg)	37.0 ± 15.5	39.4 ± 8.5	−0.484	0.633
Leg lift (kg)	89.7 ± 43.1	101.4 ± 28.0	−0.839	0.409

BMI, body mass index; SAS, Zung's Self-rating Anxiety Scale; SDNN, standard deviation of all R-R intervals; RMSSD, square root of the sum of the mean of the difference between adjacent RR intervals; LF, low frequency power; HF, high frequency power.

### Effect of resistance training on HRV in anxious female college students

Table 3 shows changes in HRV parameters as the primary outcomes after 8 weeks of resistance training. Compared with the control group, SDNN increased significantly (*P* < 0.05) and LF/HF decreased significantly (*P* < 0.05) in the intervention group. RMSSD, PNN50, LF, and HF showed an increasing trend in the intervention group, but there was no statistical difference compared to the control group.

**Table 3:** Differences of mean HRV indices compared with the control group.

**Primary outcomes**	**Intervention**		**Control**		**Post-test–pre-test**				
	**Pre-test**	**Post-test**	**Pre-test**	**Post-test**	**Intervention-control**				
	**Mean ± SD**	**Mean ± SD**	**Mean ± SD**	**Mean ± SD**	**Mean ± SD**	**95% CI**	***T*-value**	***P*-value**	**Cohen's d**
SDNN (ms)	33.9 ± 9.2	39.6 ± 12.1	36.5 ± 9.2	33.9 ± 10.8	9.4 ± 3.5	2.3,16.6	2.718	0.012	1.09
RMSSD (ms)	32.1 ± 10.9	40.5 ± 16.0	31.3 ± 12.0	30.1 ± 15.1	10.5 ± 5.8	−1.6,22.5	1.8	0.085	0.73
PNN50 (%)	12.1 ± 10.6	20.9 ± 15.3	13.8 ± 12.5	13.8 ± 16.1	9.9 ± 6.3	−3.2,22.9	1.566	0.131	0.63
LF (log)	6.2 ± 0.6	6.1 ± 0.7	6.3 ± 0.6	6.2 ± 0.8	0.01 ± 0.3	−0.6,0.6	−0.027	0.979	0.00
HF (log)	5.7 ± 0.6	6.2 ± 0.7	5.9 ± 0.8	5.5 ± 1.2	0.9 ± 0.4	−1.7, −0.2	−2.545	0.018	1.08
LF/HF	2.2 ± 1.8	1.3 ± 1.1	2.0 ± 1.5	3.2 ± 2.8	−2.2 ± 1.0	−4.3, −0.03	−2.096	0.047	0.84

BMI, body mass index; SAS, Zung's Self-rating Anxiety Scale; SDNN, standard deviation of all R-R intervals; RMSSD, square root of the sum of the mean of the difference between adjacent RR intervals; LF, low frequency power; HF, high frequency power.

### Effect of resistance training on BMI, muscle strength, and anxiety level in anxious female college students

The changes of secondary outcomes were shown in Table 4, after 8 weeks of resistance training, compared with the control group, BMI did not change significantly (*P* > 0.05), SAS score of intervention group decreased significantly (*P* < 0.05), and the 1RM of barbell bench press, Lat pull-down and leg lift were significantly increased (*P* < 0.05) in the intervention group.

**Table 4:** Differences of mean BMI, muscle strength, and anxiety level compared with the control group.

**Secondary outcomes**	**Intervention**		**Control**		**Post-test–pre-test**				
	**Pre-test**	**Post-test**	**Pre-test**	**Post-test**	**Intervention-control**				
	**Mean ± SD**	**Mean ± SD**	**Mean ± SD**	**Mean ± SD**	**Mean ± SD**	**95% CI**	***T*-value**	***P*-value**	**Cohen's d**
BMI (kg/m^2^)	21.8 ± 2.7	21.6 ± 2.6	20.7 ± 2.0	20.4 ± 1.8	−0.2 ± 0.2	−0.5, 0.2	−0.859	0.399	0.09
SAS	58.1 ± 6.2	39.0 ± 4.2	54.7 ± 6.5	46.3 ± 9.5	−10.1 ± 3.2	−16.7, −3.4	−3.12	0.005	1.26
Barbell bench press (kg)	23.8 ± 5.8	29.6 ± 7.0	22.5 ± 5.7	23.1 ± 6.7	4.4 ± 1.1	2.1, 6.8	4.006	0.001	1.70
Lat pull-down (kg)	37.0 ± 15.5	44.7 ± 12.1	39.4 ± 8.5	37.8 ± 7.9	10.5 ± 4.0	2.2, 18.7	2.638	0.015	1.09
Leg lift (kg)	89.7 ± 43.1	147.6 ± 68.3	101.4 ± 28.0	105.3 ± 31.2	54.2 ± 8.5	35.6, 72.9	6.412	0.000	2.75

BMI, body mass index; SAS, Zung's Self-rating Anxiety Scale.

## Discussion

This study shows that resistance training interventions improved HRV parameters (i.e., increments of SDNN and LF/HF ratio) during resting conditions in anxious female college students. These findings have important clinical implications to improve the autonomic nervous disorder of adolescent females with anxiety.

### Resistance training and HRV

HRV has been recognized as an effective non-invasive index reflecting autonomic nerve function. The time domain parameter SDNN of HRV mainly reflects the overall activity of autonomic nerve function. Frequency domain parameter LF is mainly mediated by sympathetic activity. RMSSD, PNN50, and HF are associated with parasympathetic activity (6). Cardiac autonomic control is an important indicator of cardiovascular health (20). Prospective longitudinal cohort studies have shown that impaired cardiac autonomic control is a strong predictor of all-cause and cardiovascular disease mortality and can be diagnosed clinically by HRV (21). Physical exercise has been proved to modulate the autonomic control of the heart (13, 22). Especially, the majority of studies on clinical populations demonstrated significant positive changes in cardiac autonomic control after resistance training (13). However, there is still a lack of such evidence-based research exploring the resistance training intervention to improve the autonomic nervous disorder of adolescent females with anxiety disorders. Our study explored that the time-domain parameter SDNN is significantly increased and the frequency-domain parameter LF/HF is significantly decreased, which indicates that resistance training can reduce the sympathetic activity and improve sympatho-vagal balance after resistance training intervention in anxious female college students.

Based on the position statements published by the American College of Sports Medicine (ACSM) and American Heart Association (AHA) (23), 70% 1RM was used as the training intensity in this study. An acute resistance training temporarily increases the sympathetic activity and temporarily decrease the parasympathetic activity; Chen, et al. found that HF drop significantly within 24 h of post-training recovery and return to baseline values by 72 h, and LF is marginally elevated in 24 h and return to normal values within 48 h (24). Therefore, in order to avoid the fatigue accumulation of autonomic nerves, we chose 72 h as the interval between the two training sessions, and resistance training twice a week as the appropriate exercise frequency in the resistance training intervention.

### Resistance training and anxiety

Resistance training has been postulated as an effective strategy to improve anxiety, especially for young patients with anxiety, and 6–8 weeks of resistance training can significantly improve anxiety (25). Gordon et al. conducted resistance training for young anxiety patients with an average age of 26.0 ± 6.2 years, twice a week, for 8 weeks, and found that their anxiety was significantly reduced (26). They also conducted resistance training intervention for obese adolescents aged 14–18 for 22 weeks, 4 times a week, and 8–15 RM, and found that their anxiety was significantly improved (27). Consistent with previous studies, we found that 8-week resistance training intervention significantly reduced the anxiety level of anxious female college students. At the same time, resistance training can bring more health benefits, such as the improvement of body composition and muscle strength (25–27). In our study, we also founded the significant increase of muscle strength, such as barbell bench press, Lat pull-down and leg lift. There was no significant change in BMI, which may be because BMI of all subjects was almost normal.

### Limitations of research

There are still some limitations in this study. The subjects of this study are female college students with anxiety, so we should be cautious when extending the results of this study to others. First of all, there are gender differences in HRV. The average heart rate of women is higher, the R-R interval is shorter, and the HRV is lower than that of men of the same age, and this difference is more obvious in young people (28). The results of this study only prove that resistance training can improve the HRV of anxious female college students, and whether the anxious male college students can get similar results by regular resistance training needs further verification. In addition, HRV is also affected by age, and it will gradually decrease with the increase of age (29). The intervention effect of resistance training is related to the basic value of HRV before intervention (13). The intervention of resistance training for anxious middle-aged and elderly women may make the HRV more improved, but it still needs to be proved by further experiments. In addition, the results of this study support the hypothesis that resistance training can have beneficial effects on HRV, it is necessary to explore the biological mechanism of resistance training affecting HRV and further clarify the intervention effect of resistance training on HRV.

In conclusion, resistance training intervention can significantly improve the anxiety level, muscle strength and HRV during resting conditions in anxious female college students. The training program in this study can be used as an exercise prescription to improve the autonomic nervous system disorder in anxious female college students.

## Data availability statement

The raw data supporting the conclusions of this article will be made available by the authors, without undue reservation.

## Ethics statement

The studies involving human participants were reviewed and approved by the Ethics Committee of Beijing Sport University. The patients/participants provided their written informed consent to participate in this study.

## Author contributions

All authors contributed to the study conception and design. Material preparation, data collection and analysis were performed by RL, RY, WC, and HR. The first draft of the manuscript was written by RL. All authors commented on previous versions of the manuscript. All authors read and approved the final manuscript.

## Funding

This study was supported by the National Key Research and Development Program of China (Nos. 2018YFC2000604 and 2020YFC2006703).

## Conflict of interest

The authors declare that the research was conducted in the absence of any commercial or financial relationships that could be construed as a potential conflict of interest.

## Publisher's note

All claims expressed in this article are solely those of the authors and do not necessarily represent those of their affiliated organizations, or those of the publisher, the editors and the reviewers. Any product that may be evaluated in this article, or claim that may be made by its manufacturer, is not guaranteed or endorsed by the publisher.
